# Pediatric chondrodermatitis nodularis chronica helicis^☆^^☆☆^

**DOI:** 10.1016/j.abd.2019.06.014

**Published:** 2020-03-19

**Authors:** Li-Wen Zhang, Lin Li, Cong-Hui Li, Wen-Ju Wang

**Affiliations:** Department of Dermatovenereology, Chengdu Second People's Hospital, Chengdu, Sichuan, China

Dear Editor,

A 9-year-old Chinese boy presented with a 2-month-history of multiple nodules affecting the right antihelix. The nodules gradually increased and were mildly painful. He was otherwise healthy. No local factors and prior skin lesions, including the history of injury to the site, were found. There was no significant medical or family history. Physical examination found four skin-colored nodules 4–5 mm on the right antihelix with a pearl necklace arrangement (Fig. 1). One of the nodules was excised and histopathologic features are shown in fig. 2. It showed irregular epidermal hyperplasia, dermal vascular endothelial cell swelling and vascular stenosis with moderate perivascular lymphocytic infiltration, and laminated fibrosis with granulomatous infiltration of epithelioid cells and lymphocytes. Blood tests including complete blood count, blood clotting index, liver and renal function, and anti-nuclear antibodies were all normal. We diagnosed this patient as having Chondrodermatitis Nodularis Chronica Helicis (CNCH) and started treatment with protective padding placed around the external ear and topical 0.5% halometasone cream. Two months later, the nodules were smaller and the pain disappeared.

**Figure 1:**
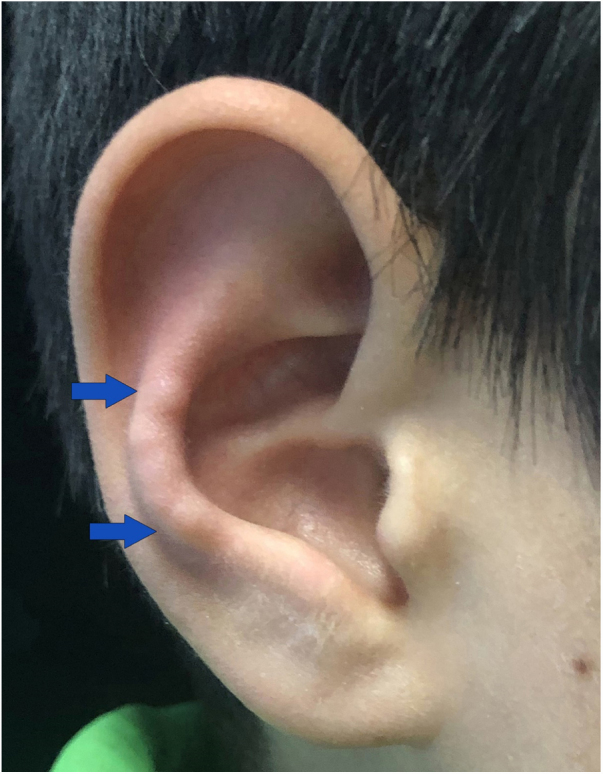
Four skin-colored nodules on the right antihelix with a pearl necklace arrangementt.

**Figure 2:**
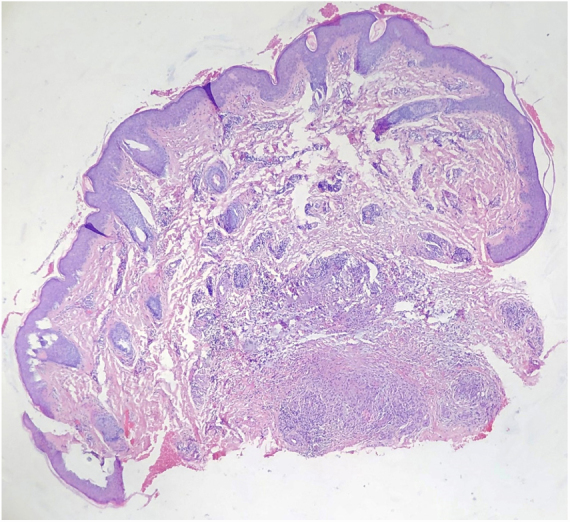
Histology showing irregular epidermal hyperplasia, dermal vascular endothelial cell swelling and vascular stenosis with moderate perivascular lymphocytic infiltration, and laminated fibrosis with granulomatous infiltration of epithelioid cells and lymphocytes (Hematoxylin & eosin, ×40).

CNCH is characterized by a benign painful erythematous nodule with a central crust, fixed to the cartilage of the helix or antihelix of the external ear. The right-sided lesions are more common than left-sided ones. CNCH most commonly occurs on the helix of the external ear in men and on the antihelix in women.^1^ It is an uncommon disorder of middle-aged and elderly individuals between 40 and 80 years, with male to female ratio of 10 to 1. Typically, CNCH presents unilaterally; however, bilateral lesions have been reported with an incidence of 3–7%.^1^ Although CNCH can occur in any age group, it has rarely been reported in children. As far as we know, our patient is the sixth pediatric case of CNCH reported by literatures (Table 1).^2^, ^3^, ^4^, ^5^ Three of these pediatric cases with CNCH reported in literature had associated connective tissue disorders. Then, some authors recommend that patients with CNCH in their fourth decade or younger should be routinely evaluated for underlying autoimmune conditions.^4^

**Table 1:** Reported cases of pediatric CNCH in the literature.

N°	Report	Age/Sex	Associated conditions	Treatment
1	Sasaki T 1999[2]	8/F	Dermatomyositis	Excision
2	Rogers NE 2003[3]	16/F	None	Excision
3	Magro CM 2005[4]	15/M	Lupus erythematosus	Not reported
4	Magro CM 2005[4]	15/M	Rheumatoid finger nodule	Not reported
5	Grigoryants V 2007[5]	10/M	None	Excision

The etiology of CNCH is unclear. The most likely cause of CNCH is ischemia or microtrauma related to sleeping on one side. Some possible contributory factors, including cartilage degeneration, ear anatomy, genetics, glomus-like vascular changes, perforating dermatoses and transepidermal elimination, pressure, autoimmune or connective tissue disorders, and trauma, may induce the onset and development of CNCH.^1^ The characteristic histopathologic features of CNCH are epidermal ulceration or a wedge-shaped epidermal defect, epithelial hyperplasia, collagen degeneration, focal fibrinoid necrosis, and inflammatory components. Cartilage may be altered, but it may frequently be normal.

The differential diagnosis of CNCH includes elastic nodule of the ear, rheumatoid nodule, calcinosis cutis, gout tophi, and glomus tumor. Elastic nodule of ear, which occurs on the anterior crus of the antihelix and produces pain simulating CNCH, occurs in a setting of chronic actinic damage. Rheumatoid nodule occurs almost exclusively in association with rheumatoid arthritis. Calcinosis cutis, the deposition of calcium in the skin and subcutaneous tissues, is classified into calciphylaxis and dystrophic, metastatic, idiopathic, and iatrogenic groups. Although the classic location of gouty tophi is the great toe, gouty tophi of the ear also is common. Glomus tumor, a rare painful vascular tumor, usually presents a solitary nodule in the distal portion of a finger, but can also occur everywhere. Generally, a biopsy is necessary to confirm the diagnosis and the histopathologic features of CNCH are characteristic.

Many treatments of CNCH are emerging; however, reoccurrence is common. Treatments can be classified into two categories. Firstly, nonsurgical procedures include carbon dioxide laser, injectable collagen implants, intralesional steroid injections, nitroglycerin gel, photodynamic therapy, removal of causative factor and relieving pressure, and topical steroids. In addition, some surgical procedures, including cartilage excision, curettage, and wedge excision, have been applied to CNCH. The first-line therapy is to relieve pressure from the site while sleeping through donut pillow or a homemade pressure-relieving device. The gold standard of therapy is surgical excision via wedge resection, despite reoccurrence if the defective cartilage is not removed.^1^ Our patient was in the early stages of CNCH without the characterized central crust in the nodule. His symptoms quickly resolved due to the early accurate diagnosis and treatment.

## Financial support

None declared.

## Authors’ contributions

Li-Wen Zhang: Approval of the final version of the manuscript; elaboration and writing of the manuscript; obtaining, analysis, and interpretation of the data; intellectual participation in the propaedeutic and/or therapeutic conduct of the studied cases; critical review of the literature.

Lin Li: Critical review of the literature.

Cong-Hui Li: Effective participation in research orientation; critical review of the manuscript.

Wen-Ju Wang: Approval of the final version of the manuscript; conception and planning of the study; effective participation in research orientation.

## Conflicts of interest

None declared.
